# HPD degradation regulated by the TTC36-STK33-PELI1 signaling axis induces tyrosinemia and neurological damage

**DOI:** 10.1038/s41467-019-12011-0

**Published:** 2019-09-19

**Authors:** Yajun Xie, Xiaoyan Lv, Dongsheng Ni, Jianing Liu, Yanxia Hu, Yamin Liu, Yunhong Liu, Rui Liu, Hui Zhao, Zhimin Lu, Qin Zhou

**Affiliations:** 1 The Ministry of Education Key Laboratory of Laboratory Medical Diagnostics, the College of Laboratory Medicine, Chongqing Medical University, 400016 Chongqing, China; 2 Department of Dermatology, West China Hospital, West China School of Medicine, Sichuan University, 610041 Chengdu, China; 3 Clinical Laboratory, The People’s Hospital of Longhua, 518109 Shenzhen, China; 4 State Key Laboratory of Oral Diseases, National Clinical Research Center for Oral Diseases, Chinese Academy of Medical Sciences Research Unit of Oral Carcinogenesis and Management, West China Hospital of Stomatology, Sichuan University, 610041 Chengdu, China; 5 Key Laboratory for Regenerative Medicine, Ministry of Education, School of Biomedical Sciences, Faculty of Medicine, The Chinese University of Hong Kong, Hong Kong, China; 6 Zhejiang Provincial Key Laboratory of Pancreatic Disease, the First Affiliated Hospital, and Institute of Translational Medicine, Zhejiang University School of Medicine, 310029 Hangzhou, China

**Keywords:** Cell biology, Ubiquitylation

## Abstract

Decreased expression of 4-hydroxyphenylpyruvic acid dioxygenase (HPD), a key enzyme for tyrosine metabolism, is a cause of human tyrosinemia. However, the regulation of HPD expression remains largely unknown. Here, we demonstrate that molecular chaperone TTC36, which is highly expressed in liver, is associated with HPD and reduces the binding of protein kinase STK33 to HPD, thereby inhibiting STK33-mediated HPD T382 phosphorylation. The reduction of HPD T382 phosphorylation results in impaired recruitment of FHA domain-containing PELI1 and PELI1-mediated HPD polyubiquitylation and degradation. Conversely, deficiency or depletion of TTC36 results in enhanced STK33-mediated HPD T382 phosphorylation and binding of PELI1 to HPD and subsequent PELI1-mediated HPD downregulation. *Ttc36*^*−/−*^ mice have reduced HPD expression in the liver and exhibit tyrosinemia, damage to hippocampal neurons, and deficits of learning and memory. These findings reveal a previously unknown regulation of HPD expression and highlight the physiological significance of TTC36-STK33-PELI1-regulated HPD expression in tyrosinemia and tyrosinemia-associated neurological disorders.

## Abstract (web summary)

Decreased expression of 4-hydroxyphenylpyruvic acid dioxygenase (HPD) has been linked to tyrosinemia, yet the mechanism underlying the regulation of HPD expression is largely unknown. Here the authors demonstrate that molecular chaperone TTC36, which is highly expressed in liver, is associated with HPD and reduces the binding of protein kinase STK33 to HPD, thereby inhibiting STK33-mediated HPD T382 phosphorylation.

## Introduction

Tyrosinemia is a metabolic disorder in which the organism fails to break down tyrosine. Symptoms include liver and kidney disturbances and cognitive disorders. Untreated hypertyrosinemia could be fatal^1^. Dysfunction of metabolic enzymes, fumarylacetoacetate hydrolase (FAH), tyrosine aminotransferase (TAT), and 4-hydroxyphenylpyruvic acid dioxygenase (HPD) in the phenylalanine and tyrosine catabolic pathway results in tyrosinemia type I, II, and III, respectively (Supplementary Fig. 1a). Elevated levels of blood tyrosine and increased amounts of urinary 4-hydroxyphenylpyruvic acid and its derivatives are common features among those with hereditary tyrosinemia^2^.

HPD catalyzes the reaction of 4-hydroxy-phenylpyruvic acid to homogentisic acid^3,4^. Homozygous mutation of the *Hpd* allele in mice blocks tyrosine catabolism^2,5–8^. Decreased activity of HPD in mouse liver causes tyrosinemia type III, which is characterized as an autosomal recessive disorder with elevated levels of blood tyrosine and massive excretion of tyrosine derivatives into the urine^5,7^. Tyrosinemia type III patients with homozygous missense mutation of *Hpd* and HPD deficiency suffer from neurological abnormalities^9,10^. Although these reports indicate a critical role of HPD in hypertyrosinemia, the mechanism underlying the regulation of HPD expression is unclear.

In this report, we demonstrate that molecular chaperone tetratricopeptide repeat domain 36 (TTC36) binds to HPD and blocks serine/threonine kinase 33 (STK33)-mediated T382 phosphorylation of HPD, thereby preventing the binding of FHA domain-containing PELI1 to HPD and subsequent HPD polyubiquitylation and degradation. Knockout of *Ttc36* in mice reduces HPD expression in liver and leads to tyrosinemia phenotypes with neuropathological changes and impaired learning and memory.

## Results

### TTC36 blocks HPD polyubiquitylation and degradation

**Fig. 1:**
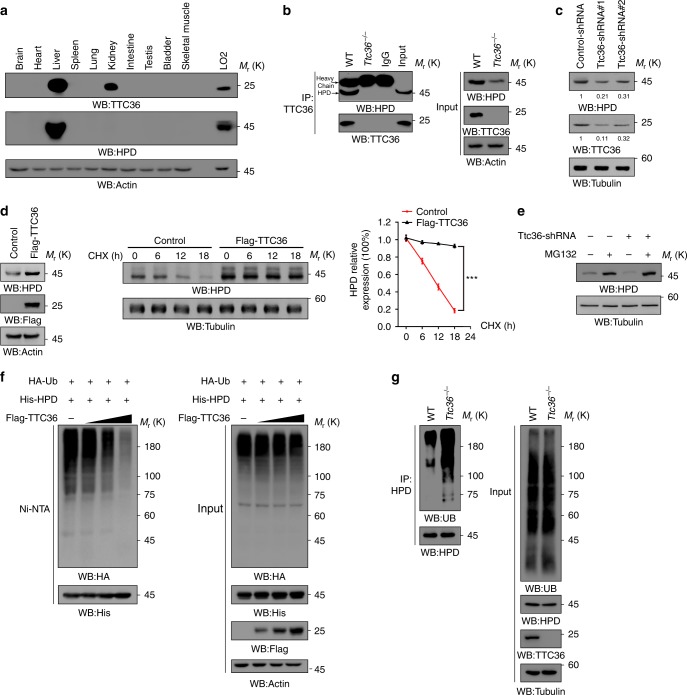
TTC36 interacts with HPD and blocks HPD polyubiquitylation and degradation. Immunoblotting analyses were performed with the indicated antibodies **a**–**g**. **a** Proteins were extracted from the indicated organs of C57BL/6 mouse. Lysates of LO2 human fetal hepatocytes were used as controls. **b** Cell lysates were extracted from mouse primary hepatocytes that isolated from the livers of wild type (WT) and *Ttc36*^*−/−*^ C57BL/6 mouse. Immunoprecipitation with an anti-TTC36 antibody were performed. **c** LO2 cells were stably transfected with a vector expressing a control shRNA or two different Ttc36-shRNAs. The quantification numbers represent relative intensity of presented bands. **d** LO2 cells with or without expressing Flag-TTC36 were prepared (left); LO2 cells with or without expressing Flag-TTC36 were treated with 10 μg mL^*−*1^ cycloheximide (CHX), and the cells were harvested at different time points (middle); quantification of HPD levels, respectively, relative to 0 h is shown (right). Data represent the means ± s.d. of three independent experiments. ***P < 0.001, based on the Student’s *t-*test. **e** LO2 cells with or without expressing Ttc36-shRNA#1were treated with or without MG-132 (10 μM) for 12 h. Immunoblotting analyses were performed with the indicated antibodies. **f** HEK293T cells were transfected with vectors expressing HA-ubiquitin, His-HPD, and TTC36 (0, 1, 2, or 4 μg). MG-132 (10 μM) was added to the cells 12 h before they were harvested with a guanidine–HCl-containing buffer. His-HPD was precipitated by the Ni-NTA beads and immunoblotting analyses were performed with the indicated antibodies. Ub ubiquitin. **g** Mouse primary hepatocytes of WT or *Ttc36*^*−/−*^ C57BL/6 mouse were treated with MG-132 (10 μM) for 12 h. The cells were harvested with denature buffer. Immunoprecipitation was performed with an anti-HPD antibody

To determine the mechanism underlying regulation of HPD expression via posttranslational modifications, we immunoprecipitated HPD from HEK293T cells. Mass spectrometry analyses revealed that molecular chaperone TTC36, a tetratricopeptide repeat (TPR) motif family member^11,12^, is an HPD-associated protein (Supplementary Fig. 1b). Immunoblotting analyses showed that TTC36 is primarily expressed in liver and moderately expressed in kidney in mice (Fig. 1a). In addition, a predominant expression of HPD in liver was also observed. Co-immunoprecipitation analyses demonstrated that HPD and TTC36 interacted with each other in wild-type (WT), but not in TTC36-deficient mouse primary hepatocytes (Fig. 1b). Expression of Flag-tagged truncated HPD mutants (Supplementary Fig. 1c) showed that VOC2 domain of HPD was critical for the association between HPD and TTC36 (Supplementary Fig. 1d). Notably, deletion of the TPR-binding domain (QEYVD) in HPD VOC2 domain had no effect on these two protein interactions (Supplementary Fig. 1e).

To determine whether TTC36 regulates HPD expression, we performed immunoblotting analyses and found that HPD expression was decreased in TTC36-deficient mouse primary hepatocytes compared to their WT counterparts (Fig. 1b, right panel). In addition, depletion of TTC36 by expressing its shRNA in LO2 cells reduced HPD expression (Fig. 1c). Consistent with these findings, Flag-TTC36 overexpression increased HPD protein expression (Fig. 1d, left panel) without altering *Hpd* mRNA levels (Supplementary Fig. 2). Furthermore, TTC36 overexpression substantially prolonged the half-life of HPD in LO2 human fetal hepatocytes treated with cycloheximide, which blocks protein translation (Fig. 1d, middle panel; quantified in Fig. 1d, right panel). These results suggest that TTC36 post-translationally stabilizes HPD expression. Treatment of LO2 cells with proteasome inhibitor MG-132 reversed the effect of TTC36 depletion on reduction of HPD expression (Fig. 1e), suggesting that TTC36 stabilizes HPD expression by blocking HPD polyubiquitylation and subsequent proteasomal degradation. This assumption was supported by evidence that overexpression of TTC36 reduced HPD polyubiquitylation in a dosage-dependent manner (Fig. 1f). In contrast, TTC36 deficiency significantly increased polyubiquitylation of HPD (Fig. 1g). These results indicate that TTC36 interacts with HPD and blocks HPD polyubiquitylation and degradation.

### TTC36 blocks the binding of PELI1 to HPD

**Fig. 2:**
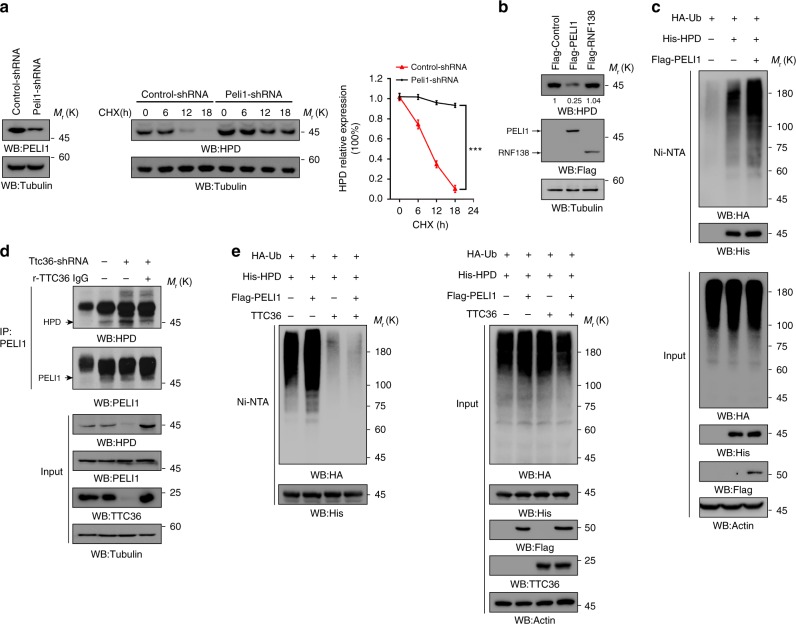
TTC36 blocks the binding of PELI1 to HPD. Immunoblotting analyses were performed with the indicated antibodies **a**–**e**. **a** LO2 cells expressing a control shRNA or Peli1-shRNA (left); LO2 cells expressing a control shRNA or Peli1-shRNA were treated with 10 μg mL^−1^ cycloheximide for 0, 6, 12, or 18 h (middle); quantification of HPD levels, respectively, relative to 0 h is shown (right). Data represent the means ± s.d. of three independent experiments. ***P < 0.001, based on the Student’s *t-*test. **b** Flag-PELI1 or Flag-RNF138 were expressed in LO2 cells. The quantification numbers represent relative intensity of presented bands. **c**, **e** HEK293T cells were transfected with the indicated plasmids and MG-132 (10 μM) was added to the cells 12 h before they were harvested with a guanidine–HCl-containing buffer. A Ni-NTA pull-down assay was performed. **d** LO2 stable cells expressing Ttc36 shRNA#1 was stably transfected with a vector expressing RNAi-resistant (r) TTC36. Immunoprecipitation analyses with an anti-PELI1 antibody were performed

Analyses of the HPD-associated protein profile, which was identified by mass spectrometry analyses, showed that PELI1 E3 ubiquitin ligase interacted with HPD (Supplementary Fig. 1b). Depletion of PELI1 by expressing its shRNA significantly enhanced the half-life of HPD in the presence of cycloheximide (Fig. 2a, middle and right panels). Conversely, overexpression of PELI1, but not RNF138 E3 ligase, reduced HPD expression (Fig. 2b). In line with these findings, overexpression of PELI1 increased HPD polyubiquitylation in HEK293T cells (Fig. 2c). These results indicate that PELI1 interacts with HPD and polyubiquitylates HPD, leading to HPD degradation.

To determine whether TTC36-alleviated HPD polyubiquitylation is due to regulation of the binding of PELI1 to HPD, we performed co-immunoprecipitation assay and found that TTC36 depletion in LO2 cells increased the interaction between endogenous PELI1 and endogenous HPD and increased degradation of HPD; these increases were abolished by reconstituted expression of RNAi-resistant (r) TTC36 (Fig. 2d). In addition, overexpression of TTC36 reduced PELI1-mediated HPD polyubiquitylation (Fig. 2e). These results strongly suggest that the interaction between TTC36 and HPD prevents the binding of PELI1 to HPD and subsequent PELI1-mediated HPD polyubiquitylation and degradation.

### PELI1 binds to phosphorylated T382 of HPD

**Fig. 3:**
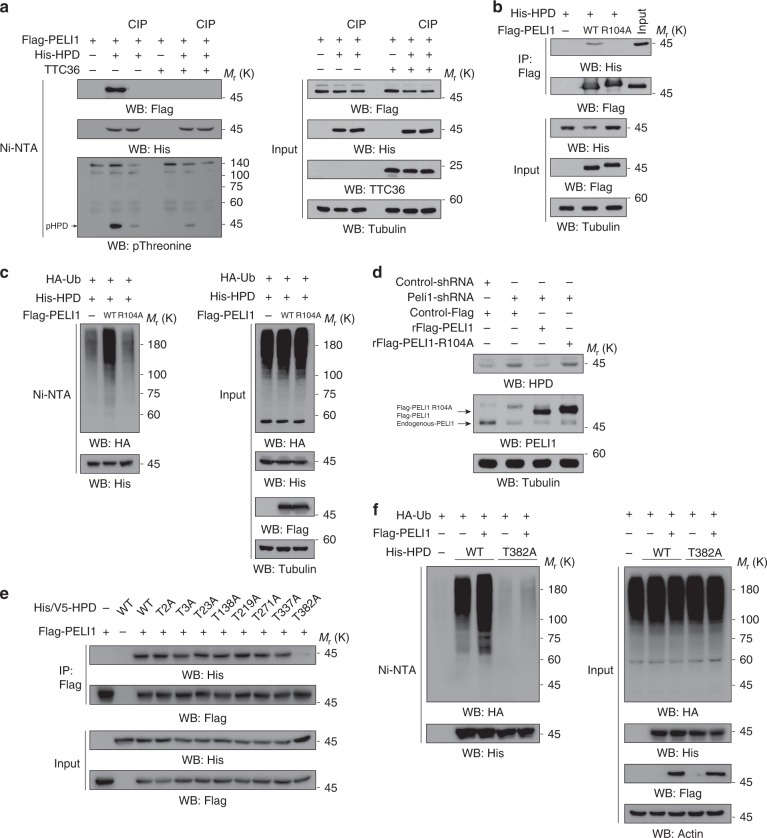
PELI1 binds to phosphorylated T382 of HPD. Immunoblotting analyses were performed with the indicated antibodies **a**–**f**. **a** HEK293T cells were transfected with the indicated plasmids. MG-132 (10 μM) was added into the culture medium of the cells 12 h before they were harvested. The cell lysates were treated with or without calf intestinal alkaline phosphatase (CIP) (1 unit CIP per µg of protein). A Ni-NTA pull-down assay was performed. **b** HEK293T cells were transfected with indicated plasmids. Immunoprecipitation with an anti-Flag antibody was performed. **c** WT Flag-PELI1 or Flag-PELI1 R104A was expressed in HEK293T cells expressing HA-Ub and His-HPD. MG-132 (10 μM) was added to the cells 12 h before they were harvested with a guanidine–HCl-containing buffer. A Ni-NTA pull-down assay was performed. **d** LO2 cells expressing Peli1 shRNA were reconstituted with expression of RNAi-resistant WT PELI1 or PELI1 R104A. **e** HEK293T cells were transfected with indicated plasmids. Immunoprecipitation with anti-Flag antibody was performed. **f** His-HPD WT or His-HPD T382A were expressed in HEK293T cells expressing HA-Ub, with or without reconstituted expression of Flag-PELI1. MG-132 (10 μM) was added to the cells 12 h before they were harvested with a guanidine–HCl-containing buffer. A Ni-NTA pull-down assay was performed

PELI1 uses its FHA domain to bind to threonine (Thr)-phosphorylated substances for ubiquitylation^13–16^. To determine whether Thr-phosphorylation of HPD is involved in the interaction between PELI1 and HPD, we immunoprecipitated HPD from HEK293T cell lysates with calf-intestinal phosphatase (CIP) treatment. CIP treatment reduced HPD Thr-phosphorylation and the binding of HPD to PELI1 (Fig. 3a). Consistent with this finding, PELI1 R104A, a PELI1 FHA domain mutant, reduced its interaction with HPD and its ability to degrade HPD compared to its wild-type (WT) counterpart (Fig. 3b). In addition, overexpression of PELI1 R104A, unlike WT PELI1, failed to induce HPD polyubiquitylation (Fig. 3c). Correspondingly, reconstituted expression of WT PELI1, but not PELI1 R104A, reduced HPD expression (Fig. 3d). These results indicate that the FHA domain of PELI1 is critical for the association of PELI1 with HPD and subsequent HPD polyubiquitylation and degradation.

To identify the phosphorylated Thr of HPD responsible for binding to PELI1, we mutated all threonine (T) residues of HPD into alanine (A). Co-immunoprecipitation analyses showed that only HPD T382A lost its binding to PELI1 (Fig. 3e). Analyses of HPD sequence showed that the region -382pTNME386- of HPD is a canonical pT*XX*E FHA domain-binding motif^17^. As expected, HPD T382A was resistant to PELI1-mediated polyubiquitylation and degradation of HPD (Fig. 3f). These results indicate that the FHA domain of PELI1 binds to phosphorylated T382 of HPD, leading to polyubiquitylation and degradation of HPD.

### TTC36 reduces STK33-mediated HPD T382 phosphorylation

**Fig. 4:**
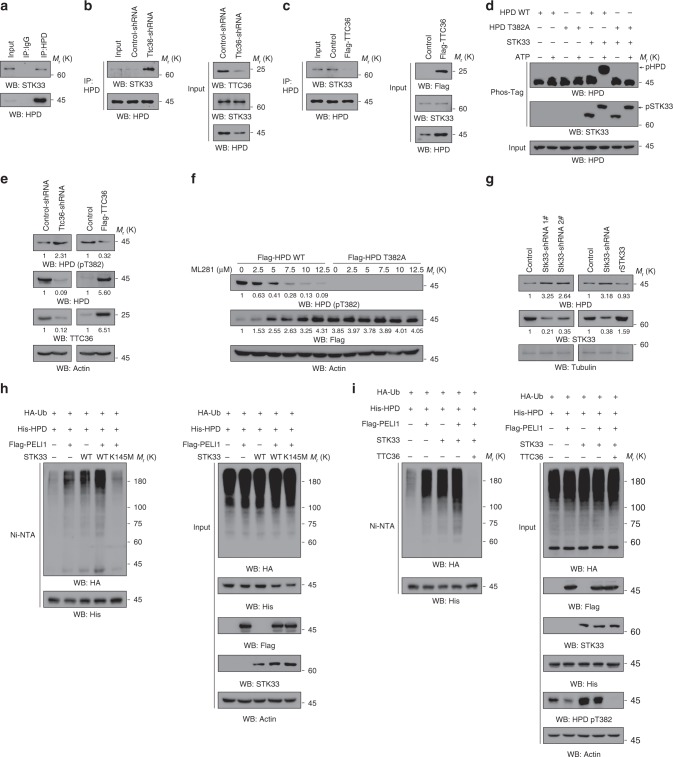
TTC36 reduces the binding of STK33 to HPD and subsequent STK33-mediated HPD T382 phosphorylation. Immunoblotting analyses were performed with the indicated antibodies **a**–**i**. **a** Immunoprecipitation analyses of LO2 cell lysates were performed with an anti-HPD antibody. **b**, **c** Ttc36 shRNA **b** or Flag-TTC36 **c** were stably expressed in LO2 cells. Immunoprecipitation analyses with an anti-HPD antibody were performed. **d** Phos-tag analysis was performed with bacterially purified His-STK33 and His-HPD or His-HPD T382A mutant in the presence or absence of ATP. **e** Ttc36 shRNA#1 (left panel) or Flag-TTC36 (right panel) were stably expressed in LO2 cells. The quantification numbers represent relative intensity of presented bands. **f** LO2 cells expressing Flag-HPD or Flag-HPD T382A mutant were treated with the indicated dosages of ML281 for 48 h. The quantification numbers represent relative intensity of presented bands. **g** LO2 cells expressing Stk33 shRNA#1 or #2 (left panel) with or without reconstituted expression of RNA-resistant(r) STK33 (right panel) were examined by immunoblotting analyses with the indicated antibodies. The quantification numbers represent relative intensity of presented bands. **h**, **i** HEK293T cells were transfected with the indicated plasmids. MG-132 (10 μM) was added 12 h before they were harvested with a guanidine–HCl-containing buffer. A Ni-NTA pull-down assay was performed

To identify the protein kinase responsible for HPD T382 phosphorylation, we analyzed the proteins associated with HPD, which were detected by mass spectrometry analyses (Supplementary Fig. 1b). We found that endogenous STK33 serine/threonine protein kinase interacted with endogenous HPD. This endogenous protein interaction was further validated by co-immunoprecipitation analyses (Fig. 4a). Notably, depletion of TTC36 increased the binding of STK33 to HPD (Fig. 4b). In contrast, overexpression of TTC36 reduced the interaction between STK33 and HPD (Fig. 4c). These results indicate that TTC36 prevents the association of STK33 with HPD.

To determine whether STK33 phosphorylates HPD, we performed an in vitro phosphorylation assay and showed that purified bacterially expressed STK33 phosphorylated purified WT HPD, but not the HPD T382A mutant (Fig. 4d). In addition, TTC36 deletion (Fig. 4e, left panel) or knockout (Supplementary Fig. 5a) increased Thr-phosphorylation of HPD, whereas TTC36 overexpression decreased Thr-phosphorylation of HPD (Fig. 4e, right panel). Treatment of LO2 cells with the STK33 inhibitor ML281 decreased Thr382-phosphorylation of HPD and increased HPD expression in LO2 cells in a dosage-dependent manner (Fig. 4f), and these alterations were abolished by the expression of HPD T382A, which remained an intact ability to bind to TTC36 (Supplementary Fig. 3). In addition, expression of two different STK33 shRNAs enhanced HPD expression in LO2 cells (Fig. 4g, left panel), and this enhancement was abrogated by reconstituted expression of STK33 (Fig. 4g, right panel). Consistent with the effect of STK33 on HPD expression, overexpression of WT STK33, but not STK33 K145M kinase-dead mutant, increased HPD polyubiquitylation (Fig. 4h). Of note, STK33-enhanced T382 phosphorylation and polyubiquitylation of HPD were blocked by overexpression of TTC36 (Fig. 4i). These results indicate that STK33 phosphorylates HPD at T382 for HPD polyubiquitylation and that TTC36 expression reduces the binding of STK33 to HPD, leading to inhibited HPD polyubiquitylation and degradation.

### TTC36 deficiency in mice results in tyrosinemia

**Fig. 5:**
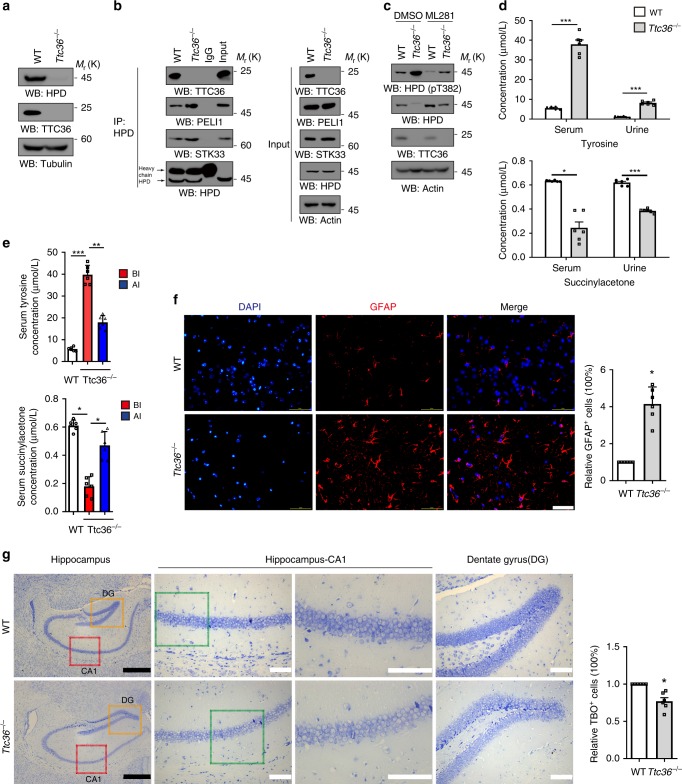
TTC36 deficiency in mice results in tyrosinemia. **a** The liver lysates of wild-type and *Ttc*36^*−*/*−*^ mice were examined by immunoblotting analyses with the indicated antibodies. **b** WT or *Ttc36*^*−/−*^ mouse primary hepatocytes were treated with MG-132 (10 μM) for 12 h and immunoprecipitation with an anti-HPD antibody. Immunoblotting analysis were performed with the indicated antibodies. **c** WT or *Ttc36*^*−/−*^ mouse primary hepatocytes were treated with ML281 (10 μM) for 48 h and immunoblotted with the indicated antibodies. **d** Serum and urine were collected from wild-type and *Ttc*36^*−*/*−*^ mice to measure the concentrations of tyrosine (upper) and succinylacetone (lower) using MS/MS methods. Data represent the means ± s.d., *n* = 6. **P *<* 0.01; ***P < 0.001. **e** Serum and urine were also collected from wild-type mice, *Ttc*36^*−*/*−*^ mice, and *Ttc*36^*−*/*−*^ mice with tail vein injection of TTC36-expressing plasmid. The concentrations of tyrosine (upper) and succinylacetone (lower) were detected using MS/MS methods. Data represent the means ± s.d., *n* = 6. **P *<* 0.01; ***P < 0.001, based on the Student’s *t-*test. BI before injection, AI after injection. **f** Immunofluorescence staining with anti-GFAP was performed on the brain cortex of wild-type and *Ttc36*^*−/−*^ mice. Scale bar = 50 μm (left); the relative percentage of GFAP-positive cells was calculated (right) and represented with the means ± s.e.m. from three independent experiments, *n* = 6. *P < 0.05, based on the Student’s *t-*test. **g** Nissl staining was performed on the hippocampus of wild-type and *Ttc36*^*−/−*^ mice. Nissl bodies were stained by toluidine blue (TBO), the areas in the CA1 and dentate gyrus regions indicted by rectangle (left) were amplified by ×20 or ×40 (middle panels for the CA1 regions (left: ×20, right: ×40), right panel for dentate gyrus regions (×20). Bar(black) = 500 μm, Bar(white) = 100 μm. The relative percentage of TBO-positive cells were quantified (right) and represented with the means ± s.e.m. of three independent experiments, *n* = 6. *P < 0.05, based on the Student’s *t-*test

To determine the physiological relevance of TTC36-enhanced HPD stability, we performed immunoblotting analyses and found that *Ttc36*^*−*/*−*^ mice had much reduced HPD expression in mouse liver tissue (Fig. 5a). In addition, *Ttc36*^*−*/*−*^ mouse primary hepatocytes had enhanced binding of HPD to STK33 and PELI1 (Fig. 5b). ML281 treatment abrogated TTC36 deficiency-enhanced HPD Thr382 phosphorylation and HPD degradation (Fig. 5c). Collectively, these results strongly suggest that TTC36 increases HPD stability by inhibiting STK33-mediated HPD phosphorylation and PELI1-dependent HPD degradation in mouse hepatocytes.

HPD deficiency has been linked to tyrosinemia and neurological disorders in human patients^9,10^. HPD deficiency in mice did not affect body weight (Supplementary Fig. 4a) nor the levels of glucose (Supplementary Fig. 4b), total triglyceride (Supplementary Figure 4c), total cholesterol (Supplementary Fig. 4d), alanine aminotransferase (ALT) (Supplementary Fig. 4e), alkaline phosphatase (ALP) (Supplementary Fig. 4f), and lactate dehydrogenase (LDHA) (Supplementary Fig. 4g) in blood and phenylalanine (Supplementary Fig. 4h) and creatinine (Supplementary Fig. 4i) in both blood and urine. However, *Ttc36*^*−*/*−*^ mice showed significantly increased tyrosine concentrations (Fig. 5d, upper panel) and decreased concentrations of succinylacetone, a downstream metabolic product of tyrosine, in both serum and urine (Fig. 5d, lower panel). These alterations were partially reverted by tail vein injection of a TTC36-expressing plasmid (Fig. 5e), and the re-expression of TTC36 in liver was detected by immunofluorescence analyses of liver tissues (Supplementary Fig. 5b, c) and immunoblotting analyses of liver lysates (Supplementary Fig. 5d). Glial fibrillary acidic protein (GFAP), which is primarily expressed in astrocyte and markedly upregulated in central nervous system (CNS) injuries^18^, had a much elevated expression level in the *Ttc36*^*−*/*−*^ mouse brain (Fig. 5f). Although hematoxylin and eosin (H&E) staining did not reveal obvious pathological changes in the brain (Supplementary Fig. 5e) and liver (Supplementary Fig. 5f), Nissl staining, which detect the function and metabolic ability of neurons^19^, showed a decreased number of Nissl bodies in neurons of the CA1 and dentate gyrus regions of *Ttc36*^*−*/*−*^ mouse hippocampus compared to its WT counterpart (Fig. 5g). These results suggest that TTC36 deficiency causes histopathological changes to mouse CNS including hippocampus.

### *Ttc36*^*−/−*^ mice exhibit impaired learning and memory

**Fig. 6:**
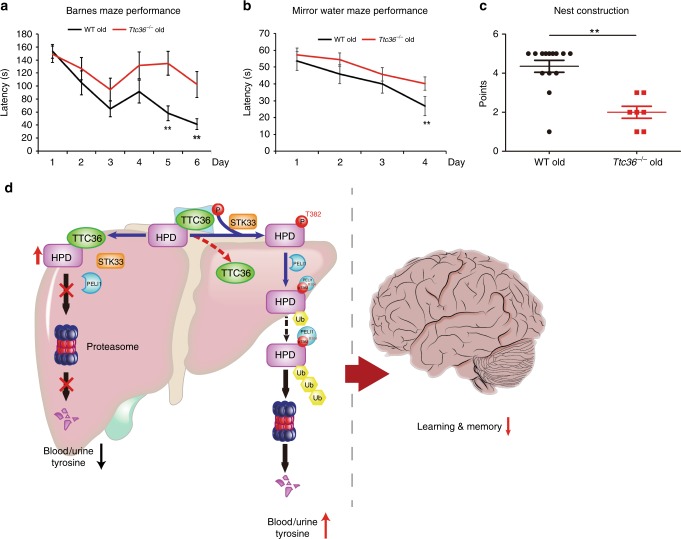
TTC36 deficiency in mice results in impaired learning and memory. **a** Mean escape latency per training block for the aged wild-type and *Ttc36*^*−/−*^ mice (8–12 months, WT group: *n* = 13, *Ttc36*^*−/−*^ group: *n* = 11), Mean ± s.e.m. **b** Mean escape latency for the aged wild-type and *Ttc36*^*−/−*^ mice (8–12 months, WT group: *n* = 13, *Ttc36*^*−/−*^ group: *n* = 10) during retention probe trial, Mean ± s.e.m. **c** Nest construction was quantified in aged wild-type and *Ttc36*^*−/−*^mice (8–12 months, WT group: *n* = 14, *Ttc36*^*−/−*^ group: *n* = 7) mice. Data from panels **a** and **c** are analyzed by two-way ANOVA with Sidak’s post-hoc test. **P < 0.01. **d** Schematic for HPD degradation regulated by the TTC36-STK33-PELI1 signaling axis in tyrosinemia, inducing neurological damage and disorders

Documented tyrosinemia Type III patients display normal function on liver, skin, or eye and some of them display neurological symptoms including developmental delay or mental retardation, or learning difficulties^9,10,19^. We next determined whether aged *Ttc36*^*−*/*−*^ mice with histopathological changes in CNS had any defects in spatial learning and memory. The Barnes Maze Test was performed to assess hippocampus-dependent spatial learning and memory. During the probe trial, *Ttc*36^*−*/*−*^ mice spent a much longer time in the target quadrant, where the escape box was formerly located, than WT mice (Fig. 6a). Similar results were also obtained by using the Mirrors Water Maze test (Fig. 6b). In addition, the Nesting Test showed that *Ttc*36^*−*/*−*^ mice scored lower than WT mice (Fig. 6c), whereas there was no difference between *Ttc*36^*−*/*−*^ mice and WT mice at a young age (Supplementary Fig. 6a, b). These results strongly suggest that TTC36 deficiency contributes to learning and memory deficits in mice.

## Discussion

Decreased expression of HPD, a key metabolic enzyme in the phenylalanine and tyrosine catabolic pathway, contributes to tyrosinemia type III, which features elevated levels of blood tyrosine, tyrosine derivatives in urine, and neurological disorders^5,7,9,10^. Here, we report a novel mechanism underlying regulation of HPD degradation. We demonstrate that TTC36 is highly expressed in liver and associates with HPD that is primarily expressed in liver. This association reduces the binding of STK33 to HPD and subsequently inhibits STK33-mediated HPD phosphorylation at T382. Phosphorylated T382 of HPD constitutes a FHA-binding motif and is recognized by FHA domain-containing PELI1 E3 ligase, leading to HPD polyubiquitylation and degradation. TTC36-mediated inhibition of HPD T382 phosphorylation thus reduces the binding of PELI1 to HPD and results in inhibition of PELI1-mediated HPD polyubiquitylation and degradation. Under conditions of deficiency or depletion of TTC36, STK33 interacts with HPD and phosphorylates HPD at T382, leading to increased recruitment of PELI1 to HPD followed by PELI1-mediated polyubiquitylation and degradation of HPD (Fig. 6d). Consistent with the TTC36-dependent upregulation of HPD in cells, Ttc36 knockout in mice reduces HPD levels in liver. Importantly, these mice exhibits tyrosinemia and pathological changes of hippocampal neurons. The deficits of learning and memory in these mice are comparable to cognitive disorders caused by hypertyrosinemia in human patients^1^. Thus, our findings reveal an instrumental mechanism underlying the physiological regulation of HPD stability and elucidate a potential cause of human tyrosinemia and tyrosinemia-associated neurological damage.

Tyrosinemia is featured by elevated tyrosine and its derivatives in serum and urine due to defected enzymes in tyrosine-catabolic pathway, such as FAH, TAT, and HPD^20^. Tyrosinemia type III resulted from mutation and genetic deficiency of HPD is a rarely recorded disease, and only 15 cases have been described^19,21,22^. Distinct from tyrosinemia type I and II, all these patients displayed no disorders of liver, kidney, skin, or eyes in neonatal period; however, nine out of the 15 patients displayed neurological symptoms after neonatal period, such as developmental delay or mental retardation, attention deficit and behavioral disturbance, absence of deep tendon reflexes, or learning difficulties^19,23^. TTC36^*−*/*−*^ mice with decreased HPD expression and corresponding tyrosinemia had a similar disease development compared to human tyrosinemia type III patients; these mice had normal liver and kidney functions and learning and memory abilities at young age, but exhibited learning and memory difficulties at old age. Thus, our findings reveal a strong correlation between human tyrosinemia type III and mouse tyrosinemia that all result from dysregulation of HPD expression. Our findings demonstrate that decreased HPD expression resulting from non-genetic mutation or deficiency of *HPD* can be a potential cause of tyrosinemia and highlight that targeting regulation of HPD stability can be an additional approach for treating tyrosinemia with reduced expression of HPD.

## Methods

### Materials

Rabbit polyclonal antibody against phosphorylated HPD T382 was obtained from Signalway Biotechnology (Pearland, TX, USA). Phosphorylated antigen peptides (RGNLpTNM) were synthesized and prepared by Zhongding bio-tech (Shanghai, China). Rabbit polyclonal antibody anti-TTC36 was produced previously^12^.

Mouse antibody against HA (sc-7392) and UB (sc-8017) were obtained from Santa Cruz Biotechnology (Santa Cruz, CA, USA). Rabbit antibodies against PELI1 (#31474S) and STK33 (#95343S), and mouse antibody against Phospho-Threonine (#9386S) were purchased from Cell Signaling Technology (Danvers, MA, USA). Rabbit anti-GFAP (SAB4501162) and anti-GAPDH (SAB2701825), mouse monoclonal anti-β-Tubulin (T5201), anti-Flag (F3165), anti-His (SAB2702219), and anti-β-Actin (A1978); and ANTI-FLAG M2 agarose affinity gel (A2220) were purchased from Sigma-Aldrich (St. Louis, MO, USA). Rabbit antibody against HPD (A6505) was obtained from Abclonal (Wuhan, China). Tyrosine Assay Kit (ab185435) and rabbit antibody against GFP (ab290) was purchased from Abcam (Cambridge, MA). Ni-NTA agarose (30210) was obtained from Qiagen (Düsseldorf, Germany). MG-132 (HY-13259), cycloheximide (CHX) (HY-12320), ML281 (HY-13495) were purchased from Med.Chem.Express (New Jersey, USA). Recombinant Human STK33 Protein (H00065975-P01) was obtained from Novus Biologicals (Missouri, USA).

### DNA constructs and mutagenesis

PCR-amplified human Hpd (NM_001171993.1), Ttc36 (NM_001080441.2), Peli1 (NM_020651.4), Stk33 (NM_001289058.1), Rnf138 (NM_001191324.1) and ubiquitin (NM_001281716.1) were subcloned into CMV, pTriEX, or pcDNA3.1 vector. pcDNA3.1-His/V5-Hpd contained nonsense mutations of T2A, T3A, T23A, T138A,

T219A, T271A, T337A, T382A. CMV-Flag-HPD contained truncation of HPD (1–280aa), ΔC (1–165aa), ΔN (166–393aa), ΔN1 (166–280aa), ΔN2 (281–393aa), and VOC2 (180–338), and ΔTPR (deletion of TPR-binding site). CMV-Flag-Peli1 contained nonsense mutation of R104A. pcDNA3.1-Stk33 contained dead mutation of K145M. All plasmids were listed in Supplementary Table 1.

For Ttc36, Peli1, and Stk33 knockdown, the shRNA pGIPZ vectors were purchased from GE Dharmacon (Lafayette, CO, USA), the target sequences were described in Supplementary Table 2.

### Mouse primary hepatocytes isolation and culture

Primary hepatocytes were isolated from 8-week-old wild type and *Ttc36*^*−/−*^ C57BL/6 mice. Briefly, mice were starved overnight before isolation. The liver was perfused through the inferior vena with 50 mL perfusion buffer A (0.1% glucose, 2.5 mM EGTA, 1% penicillin–streptomycin (HyClone), Hank’s balanced salt solution (HyClone) without Ca^2+^ and Mg^2+^) and then perfused with 50 mL perfusion buffer B (0.5 mg mL^−1^ collagenase type IV (Sigma), Hank’s balanced salt solution (HyClone) with 5 mM Ca^2+^ and 1.2 mM Mg^2+^). After perfusion, the liver was removed from the abdominal cavity and hepatocytes were released into the DMEM medium using sterile surgical scissors. Cell suspension was transferred into a 100-mesh cell filter. Hepatocytes were purified with DMEM medium at low-speed centrifugation (100 × *g*, 5 min). Viability of isolated hepatocytes was determined by trypan blue staining. Cell culture dishes for primary hepatocytes were coated with coating buffer (4% collagen type I (Corning), 0.2% glacial acetic acid in PBS). Primary hepatocytes were cultured in Hepato-medium (DMEM/F12 (Gibco) supplemented with 10% FBS, 100 nM insulin (MCE), 100 nM dexamethasone (Sangon Biotech), 2% penicillin–streptomycin (HyClone)). The primary hepatocytes were cultured at 37 °C, 5% CO_2_ in a regular incubator.

### Cell culture, lentivirus infection, and stable cell lines

The HEK293T and LO2 (No. 3142C0001000000077, National Infrastructure of Cell Line Resource, China) cells were maintained in Dulbecco’s modified Eagle’s medium (DMEM) supplemented with 10% dialyzed fetal bovine serum (HyClone) and 1% penicillin–streptomycin (HyClone). Cells were plated at a density of 4 × 10^5^ per 60-mm diameter dish 18 h prior to transfection. TTC36 overexpression, Peli1 silencing, and Stk33 silencing constructs were co-transfected in HEK293T packaging cells along with packaging, envelope, and reverse transcriptase vectors using HyFect transfection reagent (Denville Scientific, NJ, USA). For viral transduction, the cells were seeded at 50–60% confluence. Then the cells were treated overnight with the medium containing retrovirus or lentivirus harvested 48 or 72 h after transfection. Virus particles were concentrated and purified by ultra-high-speed centrifugation (25,000 × *g* for 2 h at 4 °C). LO2 cells were infected with lentivirus in the presence of 8 μg mL^−1^ polybrene (Sigma). Selection of stable clones was carried out using puromycin (Invivogen).

### Immunoprecipitation and immunoblotting analysis

Immunoprecipitation and immunoblotting analysis were performed according to X. Li et al. ^24^. Extraction of proteins from cultured HEK293T cells, LO2 cells, and mouse primary hepatocytes were performed with a lysis buffer (50 mM Tris–HCl pH 7.5, 0.1% SDS, 1% Triton X-100,150 mM NaCl, 1 mM dithiothreitol, 0.5 mM EDTA, 100 mM PMSF, 100 mM leupeptin, 1 mM aprotinin, 100 mM sodium orthovanadate, 100 mM sodium pyrophosphate, and 1 mM sodium fluoride). Cell extracts were clarified by centrifugation at 13,400 × *g*, and the supernatants (2 mg protein mL^−1^) were subjected to immunoprecipitation with the indicated antibodies. After overnight incubation at 4 °C, protein A agarose beads were added and left for an additional 3 h. Immunocomplexes were washed with lysis buffer three times and then subjected to immunoblotting analyses with corresponding antibodies and supplementary Table 1. The band intensity was quantified using the Image Lab software program (Bio-Rad).

### Mass spectrometry analysis

Mass spectrometry analyses were performed according to the report of Y. Jiang et al. ^25^. In brief, immunoprecipitation products were separated by SDS–PAGE and stained with Coomassie Brilliant Blue. Visible protein bands were excised from SDS–PAGE and decolored with gradient mixture of acetonitrile and 50 mM ammonium bicarbonate buffer at room temperature. When all blue completely disappears, protein bands were digested in gel with 200 ng of modified sequencing-grade trypsin (Promega) dissolved in 50 mM ammonium bicarbonate buffer containing RapiGest (Waters Corporation) overnight at 37 °C. The digested samples were analyzed using high-sensitivity liquid chromatography tandem mass spectrometry with an Orbitrap Elite mass spectrometer (Thermo Fisher Scientific). Proteins were identified by searching the fragment spectra against the Swiss-Prot protein database (EBI) using the Mascot search engine (version 2.3; Matrix Science) with the Proteome Discoverer software program (version 1.4; Thermo Fisher Scientific).

### RNA extraction and q-PCR

Total RNA was extracted from mouse liver tissue using TRIzol reagent (Invitrogen) according to manual. For each sample, 1 μm of the total RNA was used for first cDNA synthesis in a 20 μL reaction system using an iScript cDNA synthesis kit (Bio-Rad). cDNA library(1 μL) was used for each sample in a 25 μL PCR reaction. Fast SYBR Green Master Mix (Bio-Rad) was used to determine the threshold cycle (Ct) value of each sample using a CFX96 real-time PCR detection system (Bio-Rad). 18S served as the normalization gene in these studies. The relative expression levels for the target genes were presented as 2DCt (the Ct of 18S minus the Ct of the target gene). Primer sequences were listed in supplementary Table 3.

### Ubiquitination assay by Ni-NTA resin

HEK293T cells were transfected with the indicated plasmids for 48 h and lysed using the denatured buffer (6 M guanidine–HCl pH 8.0, 0.1 M Na_2_HPO_4_/NaH_2_PO_4_, and 10 mM imidazole) containing 5 mM N-ethylmaleimide to prevent deubiquitylation. Ni-NTA His-binding resin were incubated with cell lysate for 12 h at 4 °C. The beads were washed with the lysis buffer for three times. Immunoblotting was performed using an anti-HA antibody. Internal His-HPD and HA-Ub were detected with anti-His and anti-HA antibody. Expression of the β-Actin protein was used as a loading control.

### Ubiquitination assay by immunoprecipitation

Endogenous protein ubiquitination assays were performed by immunoprecipitation^26^. Primary mouse hepatocytes were lysed with 1% SDS buffer (50 mM Tris–HCl pH 7.5, 150 mM NaCl, 1% SDS, and 10 mM DTT) and denatured by heating for 30 min. The lysates were diluted with lysis buffer (50 mM Tris–HCl pH 7.5, 150 mM NaCl, 1 mM EDTA, and 1% Triton X-100), so that the concentration of SDS was decreased to 0.1%. The diluted lysates were immunoprecipitated with an anti-ubiquitin antibody (Santa Cruz Biotechnology, sc-8017) overnight at 4 °C, which was followed by adding protein A agarose to the lysates for 2 h. Immunocomplexes were washed with lysis buffer three times and then subjected to immunoblotting analyses.

### His pull-down assay

HEK293T cells were transfected with the indicated plasmids for 48 h and treated with DMSO or MG-132 for 12 h^27^. Then cells were harvested in lysis buffer and some cell lysate were incubated with alkaline phosphatase, calf intestinal (CIP) (New England Biolabs) (1 unit per μg protein) for 60 min at 37 °C. Ni-NTA His-binding resin were incubated with cell lysate for 12 h at 4 °C^16^. The beads were washed with lysis buffer three times. Immunoblotting analyses were performed with the indicated antibodies.

### Recombinant protein purification

Wild-type His-HPD and mutant His-HPD T382A were expressed in bacteria. Bacterial cultures were grown at 37 °C to an OD600 of ~0.6 before inducing with 0.1 mM IPTG for 3 h. Cell pellets were collected, and resuspended in 9 mL lysis buffer (50 mM Tris–HCl pH 8.0, 120 mM NaCl, 1 mM DTT, 1 mM PMSF, 1 mM benzamidine, 20 μg mL^−1^ leupeptin, and 1.0 μg mL^−1^ aprotinin), and lysed by sonication. Triton X-100 was added to the lysates at a final concentration of 1% and left on ice for 20 min, before centrifugation at 13,400 × *g* for 10 min (4 °C). Cleared lysates were then bound to Ni-NTA agarose for 4 h, with rolling at 4 °C. Beads were washed extensively with lysis buffer before eluting for 20 min in an elution buffer (0.5 M imidazole, 0.5 M NaCl, 20 mM Tris–HCl pH 7.9). Eluted proteins were then dialyzed extensively against 20 mM Tris–HCl pH 8.0, 50 mM NaCl, 10% glycerol, and 1 mM DTT^25^.

### Phos-tag analysis

Phos-tag analysis was performed according to the manufacturer’s instructions. The phosphorylation reactions were done by mixing 0.3 μg purified His-STK33 and 1 μg His-HPD or His-HPD T382A mutant in 20 μL kinase assay buffer containing 10 mM ATP, 25 mM MgCl_2_, and 20 mM Tris–HCl (pH7.4) for 60 min at 30 °C^25,28,29^. Reactions were stopped by adding an equal volume of 2 × SDS–polyacrylamide gel electrophoresis (PAGE) sample buffer and boiling for 5 min. Samples were then separated by 8% SDS–PAGE with 50 μM Phos-binding reagent acrylamide (APExBIO, F4002) and transferred onto PVDF membranes for immunoblotting analyses^30^.

### Animal studies and tail vein plasmid injection

All experiments were carried out using 6–8-week-old C57BL/6 male mice as the young group and 8–12-month-old male mice as the old group. Animals were housed under standard laboratory conditions (12:12 h light–dark cycle with lights on at 07.00 a.m., 23 °C temperature, and 40–50% relative humidity), food and water were provided ad libitum. Five mice were housed per cage. All mouse surgeries were performed under sodium pentobarbital anesthesia. Animals were treated according to the recommendations in the Guide for the Care and Use of Laboratory Animals of Chongqing Medical University. The experimental procedures were approved by the Animal Experimental Ethics Committee of Chongqing Medical University.

Plasmids DNA was injected into mice through tail vein with hydrodynamics-based transfection^31^. Briefly, 8–10-month-old male WT and *Ttc36*^*−/−*^ mice were randomly divided into three groups, as wild type group, before injection group, and after injection group, and 50 μg endotoxin-free pcDNA3.1-*Ttc36* dissolved in sterile saline (the volume of saline was 10% the body weight) was injected into the tail vein in 7 s at room temperature. Mice were sacrificed at 72 h after the plasmid injection.

### Tyrosine detection

Tyrosine Assay Kit (Abcam, ab185435) was used for serum tyrosine detection following the manufacturer’s instructions. In brief, tyrosine assay reagent, standard curve, control, and samples were prepared in a 96-well plate and reaction occurred at room temperature for 60 min protected from light. Then plate was measured for absorbance at OD492 nm in a microplate reader.

### Biochemical and metabolic analyses

Blood was collected from 10 to 12-week-old male WT and *Ttc36*^*−/−*^ mice^32^. In compliance with the laboratory animal care guidelines, ~0.4 mL of blood per mouse was collected. Serum was separated and analyzed for glucose, triglyceride, cholesterol, ALT, ALP, and LDHA, and creatinine in blood and urine by automatic biochemical analyzer (IDEXX Catalyst One, USA) using commercial kits and following the manufacturer’s instruction. Urine was collected over 24 h using metabolic cages (PhenoMaster-R/M, German).

Urine and serum succinylacetone were analyzed by MS/MS from dried blood spot (DBS)^33^. All blood pools were spotted onto filter paper and dried overnight at ambient temperature. A single 1/8″ (3.2 mm) diameter filter paper, respectively, placed into 96-well polypropylene microtiter plates was extracted with 100 µL of methanolic solution for 30 min at ambient temperature with gentle agitation. Then, 100 µL 80:20% acetonitrile:water containing 0.1% formic acid, 15 mmol L^−1^ hydrazine hydrate (0.1% by volume), and 100 nmol L^−1^ of the internal standard, ^13^C_5_-SUA were added to the residual paper punch. The plate was covered with aluminum foil to avoid evaporation and incubated for 45 min at 37 °C with gentle agitation. After incubation, the extraction was transferred to another 96-well plate and dried under gentle flow of nitrogen (50 °C, 15 min). Methanol (50 µL) was added to each well, agitated, and evaporated under nitrogen flow (50 °C, 10 min) for removing residual hydrazine. The plate was washed using the following steps, i.e. the addition of 100 µL of fresh methanol, agitating it, and finally by removing washed fluid. And the washing step took no more than 5 min for one full plate. The washed plate was then subjected to derivatization for SUAC analysis.

### Haematoxylin and eosin (H&E) staining and Nissl staining

The brain and liver of each mouse was harvested, fixed in 4% formaldehyde, and embedded in paraffin. For the H&E staining, the 4 µm sections were dewaxed and rehydrated with an ethanol series; and stained with haematoxylin (2 min), decolorized with acid alcohol (35 s), immersed with lithium carbonate (55 s), and 80% ethanol (1 min), and counterstained with eosin. For the Nissl staining, the 5 µm sections were dyed with Nissl dye, which was pre-heated at 60 °C for 20 min, rinsed with distilled water, and differentiated and decolorized with 95% ethanol until the background is clear. Finally, the sections were dehydrated with ethanol, made to be transparent with xylene and sealed. Images were captured with a Leica DM4B microscope (Leica, Wetzlar, Germany). Photographs were taken with a DFC550 Leica camera (Leica, Wetzlar, Germany). For quantitative analyses, three slides of each organ with at least three representative high power fields (HPF, ×40) per slide were evaluated in a blinded fashion.

### Immunofluorescence analyses

Sectioned brain and liver (4 μm paraffin) were prepared for immuno-fluorescence by dewaxing and rehydration with an ethanol series using standard protocols^34^. Primary antibodies: anti-GFAP (Sigma, SAB4501162), anti-TTC36 (produced previously^12^) was diluted (1:100) in 1% heat-inactivated sheep serum in PBS and sections incubated overnight at 4 °C. Tissues were incubated with fluorescently labeled secondary antibodies for 60 min at room temperature followed by 5 min with DAPI to label nuclei. Sections were photographed using a Leica TCS SP8 confocal microscope (Leica, Wetzlar, Germany). For quantitative analyses, three slides of each organ with at least three representative high power fields (HPF, ×40) per slide were evaluated in a blinded fashion.

### Morris water maze test (MWM)

The MWM was performed to examine the spatial memory of the mice with or without knockout *of TTC36*^35,36^. In brief, MWM was conducted in a round and black water tank with 153 cm in diameter and 63 cm in height. This tank was filled with water at 26 °C to 53 cm in depth. A black escape platform with 10.8 cm in diameter and 1.5 cm beneath the surface of the water in height was set up at a fixed position throughout the testing period. The digital tracking system was placed on the top of water tank to record the trial. During the trials, the mice were placed into the water at a starting position towards the wall of the pool. These mice had to swim and find the escape platform by different cues on the wall of pool or the surroundings. Each mouse was tested four times (60 s each) in a day and 4–6 days in total. The order of starting positions is random by trials, but constant in each session. If the mouse, who had failed to find the platform within 90 s, was guided to it. At the end of each trial, the mouse stayed on the platform for 30 s. The path and time that they found the escape platform were recorded for each trial and registered as latency in seconds. In each session, the latency of first trial was recorded as reference memory, and the average latency of four trials was used for working memory.

### Barnes Maze test

Barnes Maze (BM) was performed by using BM apparatus (Panlab-Harvard Apparatus, Spain) to evaluate the spatial learning and memory of the mice with or without knockout *of TTC36*^37^. This apparatus consists of Barnes labyrinth disc (120 cm in diameter), a video device, and one computer. The surface of Barnes labyrinth disc was equipped with 18 holes around the perimeter, and a hidden drawer-like dark box (escape chamber) was set up under one of the holes. Some visual cues around the maze were placed to facilitate learning; light (600 lx) or a fan was located around the maze as the weak aversive stimulus to motivate mice to escape from the platform. The day before experiment, the mouse was placed in the escape box from the target hole for 240 s. During the test period, the mouse was covered with an opaque box in the middle of the disc. After 10 s, the mouse was released, and the software was automatically started. If the mouse found the target hole within 180 s, the software was automatically stopped and recorded the stopping time as its latency. If the mouse failed to find the target hole within 180 s, the software was stopped at 180 s, which was recorded as the latency of the mouse. The mice who had failed to find the escape chamber within 180 s was guided to the escape chamber. The mice were allowed to stay in escape chamber for 2 min in each trial. After each trial, the disc was cleaned with 70% ethanol to avoid any odor cues. Each mouse was subjected to four trials a day with 15 min intervals between each trial. The location of the escape chamber varied from one to another.

### Nesting test

Both male and female mice will build a nest when they are provided with suitable building material. This species-specific behavior requires organization of a complex set of behaviors, and appears highly sensitive to interventions or mutations that affect hippocampal function. To assess nest building behavior, additional nesting material is introduced into each animal’s home-cage. The next morning, nest quality is graded into 1–5 rating scale^38^. To assess nest-building behavior, additional nesting material (Nestlet of 3 g compressed cotton) was introduced into each animal’s home-cage ~3 h before the start of the dark phase. The next morning, nest-building behavior was scored according to a rating scale of 1–5^38^: 1 = Nestlet > 90% intact, 2 = Nestlet 50–90% intact, 3 = Nestlet mostly shredded but no identifiable nest site, 4 = identifiable but flat nest, and 5 = crater-shaped nest.

### Statistical analysis

Measurements were taken from distinct samples. Unless noted, all of the experiments were repeated at least three times. The quantitative data except mice behavioral analysis data were presented as the mean ± s.d. A Two-tailed paired or unpaired *t*-test statistical analysis was performed using GraphPad Prism 8 software (GraphPad, San Diego, CA, USA). *P*-value < 0.05 and <0.01 means significance and strong significance, respectively. Data of mice behavioral analysis were present as the mean ± s.e.m. and the two-way ANOVA were used for statistical test. The statistical significance was *P* < 0.05. Western blots were analyzed using Image J (NIH, Bethesda, MD, USA) or Quantity one (Bio-Rad, Berkley, CA, USA). Imaging data were analyzed using Leica LAS-X Image Analysis (Leica, Wetzlar, Germany) and quantified data were shown as mean ± s.e.m.

### Reporting summary

Further information on research design is available in the Nature Research Reporting Summary linked to this article.

## Supplementary information

Supplementary Information

Reporting Summary

Source Data

## Data Availability

All relevant data are available upon reasonable request. The source data underlying Figs. 1a–g, 2a–e, 3a–f, 4a–i, 5a–c and Supplementary Figs. 1d, 1e, 3, and 5a are provided as a Source Data File. Figure 6d is completely created by our co-authors, no previously created elements are involved in this figure.
