# Supplementary material for: HPD degradation regulated by the TTC36-STK33-PELI1 signaling axis induces tyrosinemia and neurological damage
Source: Nat Commun. 2019 Sep 19;10:4266. doi: 10.1038/s41467-019-12011-0 (PMC6753076; doi:10.1038/s41467-019-12011-0)
Supplement: Supplementary file 1 — Supplementary Information [file 41467_2019_12011_MOESM1_ESM.pdf]

Supplementary Information

HPD degradation regulated by the TTC36-STK33-PELI1 signaling axis induces tyrosinemia and neurological damage

Yajun Xie, Xiaoyan Lv, Dongsheng Ni, Jianing Liu, Yanxia Hu, Yamin Liu et al.

Contents:

1. **Supplementary Figure 1.** TTC36 interacts with HPD and increases HPD expression.
2. **Supplementary Figure 2.** TTC36 does not affect *Hpd* mRNA level.
3. **Supplementary Figure 3.** HPD T382A does not affect the interaction of TTC36 and HPD.
4. **Supplementary Figure 4.** TTC36 deficiency in mice results in tyrosinemia.
5. **Supplementary Figure 5.** TTC36 deficiency in mice increased HPD expression.
6. **Supplementary Figure 6.** TTC36 deficiency in mice impaired learning and memory.
7. **Supplementary Table 1.** Key resources for this paper.
8. **Supplementary Table 2.** Target sequences used for shRNA construction.
9. **Supplementary Table 3.** Primers used for quantitative PCR.

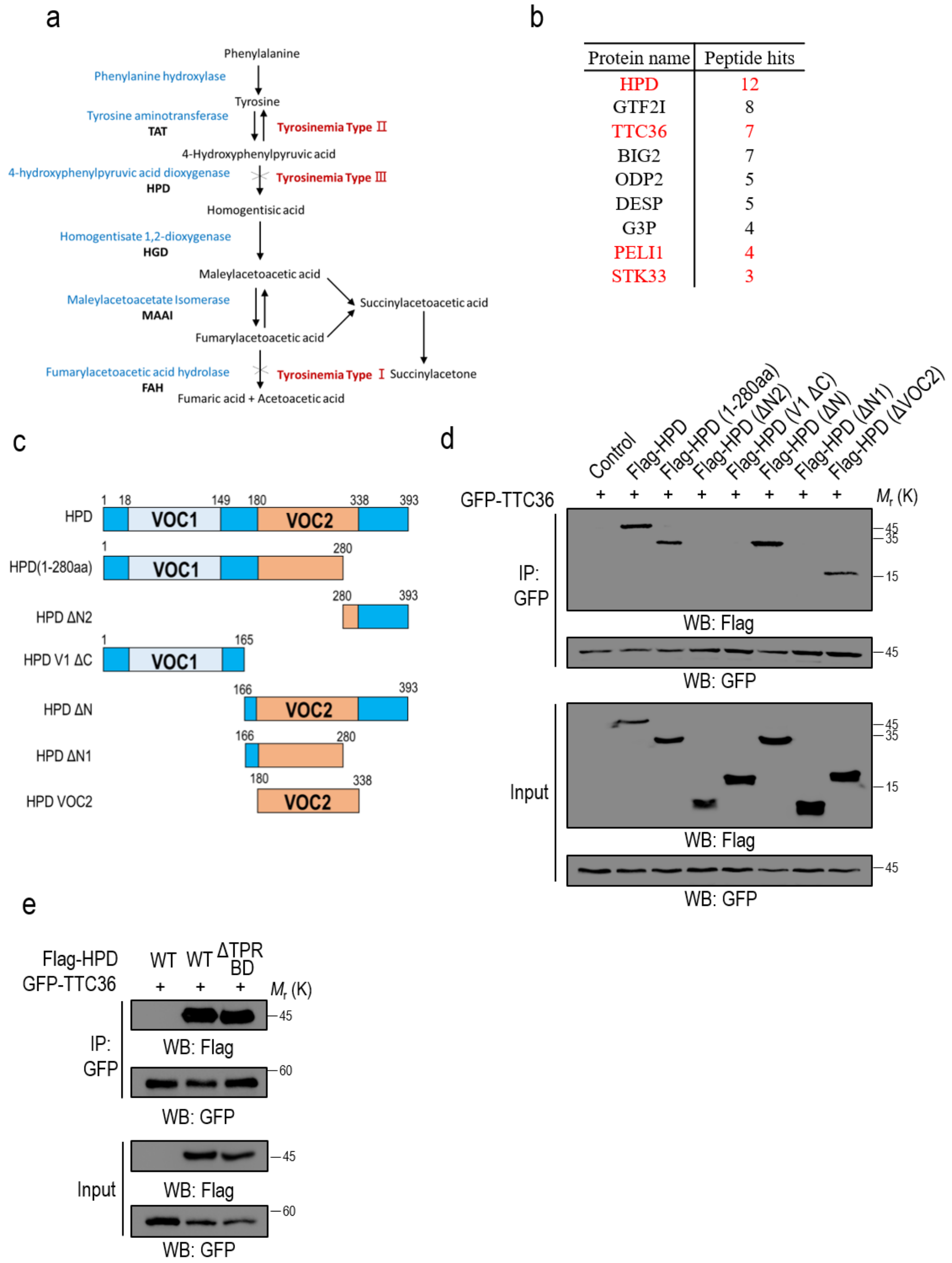

Protein name	Peptide hits
HPD	12
GTF2I	8
TTC36	7
BIG2	7
ODP2	5
DESP	5
G3P	4
PELI1	4
STK33	3

Supplementary Figure 1. TTC36 interacts with HPD and increases HPD expression. a, Schematic presentation of the tyrosine metabolism pathway. b, The cell lysates of HEK293T cells with expression of GFP-HPD was immunoprecipitated with an anti-GFP antibody and analyzed by mass spectrometry. The identified proteins with the peptide hits were shown. c, Schematic presentation of HPD truncations. The Flag-tagged HPD truncations were used for TTC36-HPD binding domain screen. d, HEK293T cells were transfected with the indicated plasmids. Immunoprecipitation with anti-GFP antibody was performed and analyzed by immunoblotting with the indicated antibodies. e, HEK293T cells were transfected with the indicated plasmids. Immunoprecipitation with anti-GFP antibody was performed and analyzed by immunoblotting with the indicated antibodies.

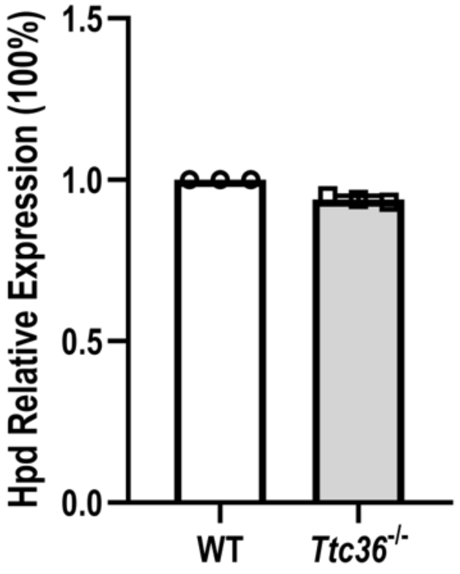

Supplementary Figure 2. TTC36 does not affect *Hpd* mRNA level. mRNA levels of *Hpd* in the livers of wild-type and *Ttc*36$^{-/-}$ mice were determined by real-time PCR. The data are presented as means ± s.d. from triplicate samples. * $P < 0.05$, based on the Student's *t* test.

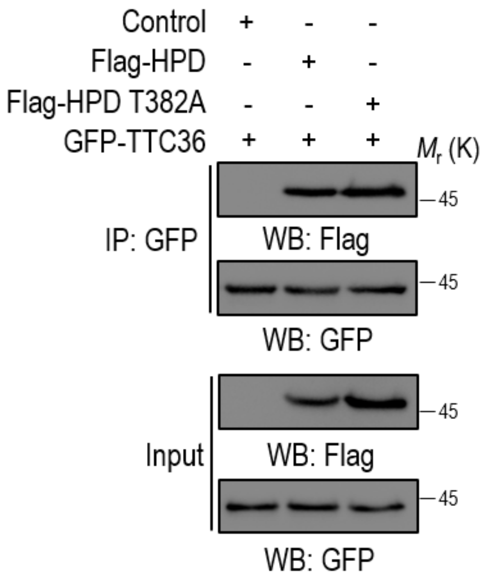

Supplementary Figure 3. HPD T382A does not affect the interaction of TTC36 and HPD. HEK293T cells with GFP-TTC36 expression were transfected with WT HPD or T382A mutant. The immunoprecipitation and immunoblotting analyses were performed with the indicated antibodies**.**

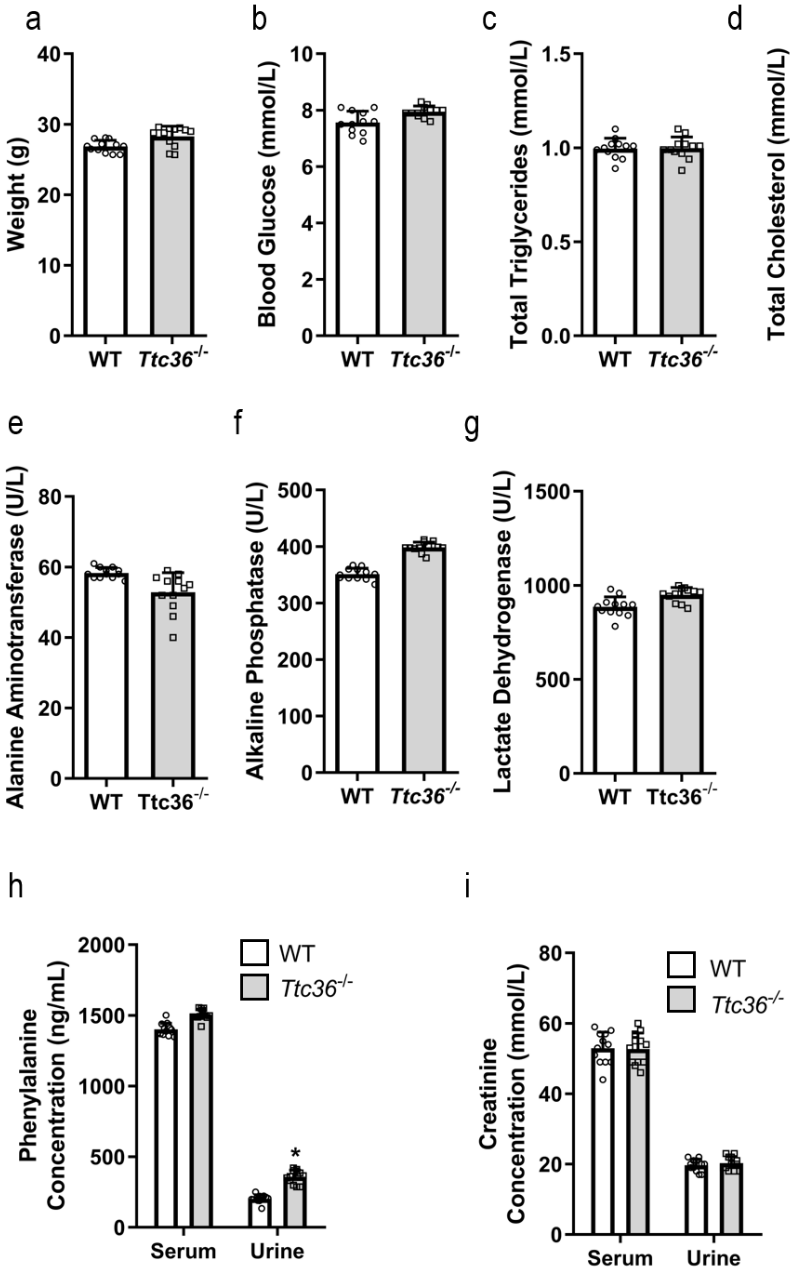

Supplementary Figure 4. TTC36 deficiency in mice results in tyrosinemia. a, Body weights of wild-type and *Ttc36*$^{-/-}$ mice (10-12 weeks, n=12) were measured. b, Blood glucose concentrations of wild-type and *Ttc36*$^{-/-}$ mice (10-12 weeks, n=12) were measured by automatic biochemical analyzer. c-i, Triglyceride (c), cholesterol (d), alanine aminotransferase (ALT) (e), alkaline phosphatase (ALP) (f), and lactate dehydrogenase (LDHA) (g) in blood and phenylalanine (h) and creatinine (i) in both blood and urine of wild-type and *Ttc36*$^{-/-}$ mice (10-12 weeks, n=12) were measured by automatic biochemical analyzer. Data represent the means ± s.d. of three independent experiments. * $P < 0.05$, based on the Student's *t* test.

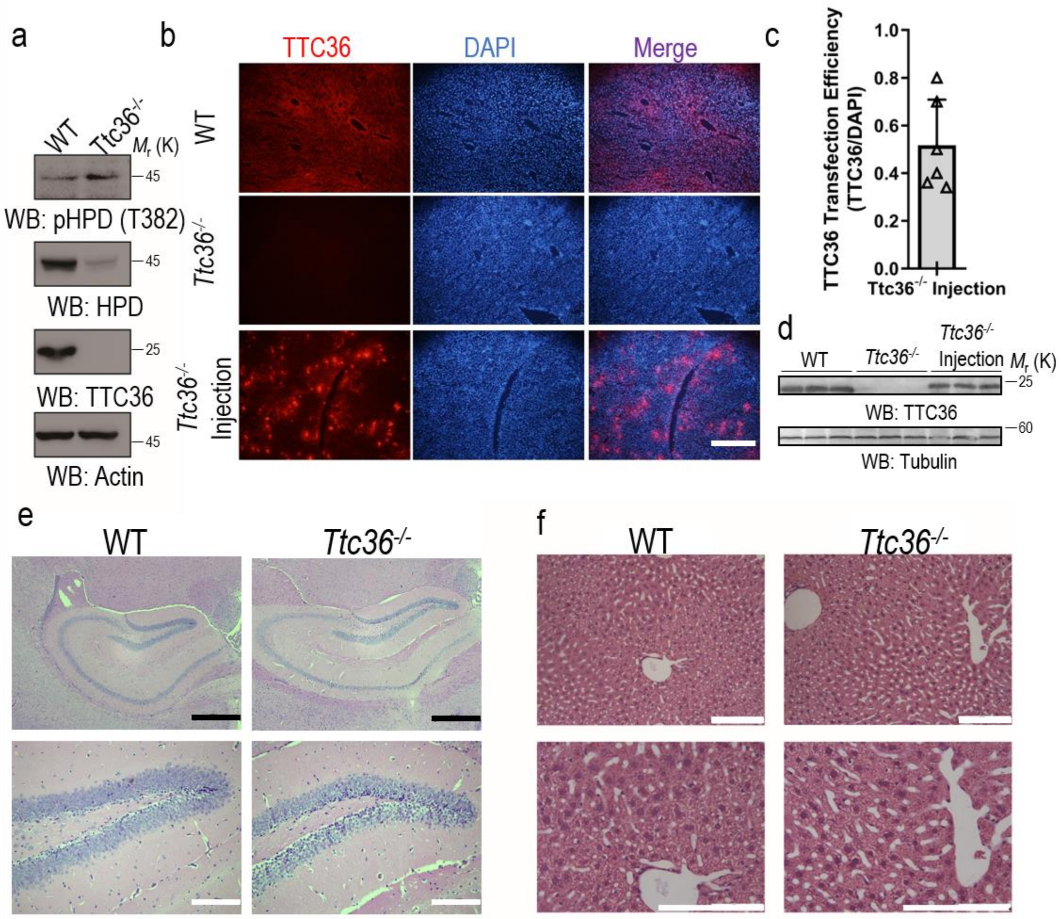

Supplementary Figure 5. TTC36 deficiency in mice increased HPD expression. a, The livers of wild-type and *Ttc*36$^{-/-}$ mice were examined by immunoblotting analyses with the indicated antibodies. b-d, Immunofluorescence staining with an anti-TTC36 was carried out on the livers of wild-type mice, *Ttc36*$^{-/-}$ mice, and *Ttc36*$^{-/-}$ mice with tail vein-injected TTC36 overexpression plasmid. Bar = 500 μm. The percentages of TTC36 expressed cells in *Ttc36*$^{-/-}$ mice liver were calculated (c), data represent the means ± s.d. from triplicate samples. Immunoblotting analyses of the liver lysates were performed with the indicated antibodies. e, Hematoxylin and eosin staining analysis of hippocampus of wild-type and *Ttc*36$^{-/-}$ mice. Bar(black)=500μm; Bar(white)=100μm. f, Hematoxylin and eosin staining analysis of livers of wild-type and *Ttc*36$^{-/-}$ mice. Bar=100μm.

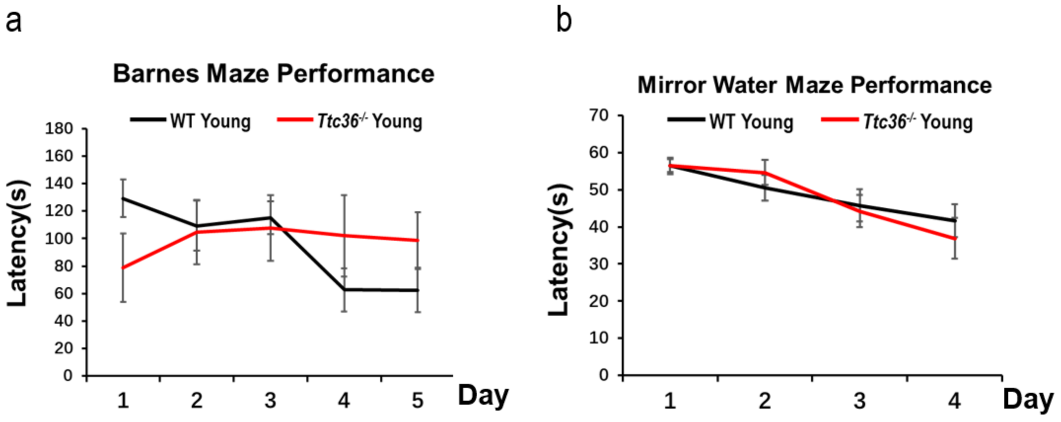

Supplementary Figure 6. TTC36 deficiency in mice impaired learning and memory. a, Latency time of young wild-type and *Ttc*36$^{-/-}$ mice in Barnes Maze Test, Means ± s.e.m. (6-8 weeks, n ≥8 per group). b, Latency time of young wild-type and *Ttc*36$^{-/-}$ mice in Mirrors Water Maze Test, Means ± s.e.m. (6-8 weeks, n ≥6 per group).

Supplementary Table 1. Key resources for this paper.

**Chemicals**			
**REAGENT or RESOURCE**	**SOURCE**	**LOCATION**	**IDENTIFIER**
Fetal bovine serum	Biological Industries	Kibbutz Beit-Haemek, Israel	Cat#04-001-1A
Dulbecco's modified Eagle's medium (DMEM)	Gibco (by Thermo)	New York, USA	Cat#11965092
Lipofectamine™ 2000 Transfection Reagent	Invitrogen	California, USA	Cat#11668019
Penicillin-Streptomycin 100X solution	Hyclone	Utah, USA	Cat#SV30010
Hank's Balanced Salt Solution	Hyclone	Utah, USA	Cat#SH30031.02
TRIzol	Invitrogen	California, USA	Cat#15596026
SYBR Green Master Mix	Bio-Rad	California, USA	Cat#1708884
Polybrene (hexadimethrine bromide)	Sigma-Aldrich	Missouri, USA	Cat#107689
puromycin	Invivogen	China, Hong Kong	Cat#ant-pr
collagenase type IV	Sigma-Aldrich	Missouri, USA	Cat#C5138
collagen type I	CORNING Life sciences	New York, USA	Cat#354249
protein A agarose	GE healthcare	Utah, USA	Cat#17-0780-01
ANTI-FLAG M2 agarose affinity gel	Sigma-Aldrich	Missouri, USA	Cat#A2220
Ni-NTA agarose	Qiagen	Düsseldorf, Germany	Cat#30210
MG-132	MedChemExpress	New Jersey, USA	Cat#HY-13259
Cycloheximide(CHX)	MedChemExpress	New Jersey, USA	Cat#HY-12320
ML281	MedChemExpress	New Jersey, USA	Cat#HY-13495
Insulin(human)	MedChemExpress	New Jersey, USA	Cat#HY-P0035
DAPI	Sigma-Aldrich	Missouri, USA	Cat#D9542;
guanidine HCl	Sigma-Aldrich	Missouri, USA	Cat#V900385
Phenylmethylsulfonyl fluoride (PMSF)	Sigma-Aldrich	Missouri, USA	Cat#52332;
Bovine Serum Albumin	Sigma-Aldrich	Missouri, USA	Cat#B2064
Diaminobenzidine	Sigma-Aldrich	Missouri, USA	Cat#D12384
Recombinant Human STK33 Protein	Novus Biologicals	Missouri, USA	Cat#H00065975-P01
Tyrosine Assay Kit	Abcam	Massachasetls, USA	Cat#ab185435
Dexamethasone	Sangon Biotech	Shanghai, China	Cat#A601187
HyFect transfection reagent	Denville Scientific	New Jersey,USA	Cat#TAMB33L
Alkaline Phosphatase, Calf Intestinal (CIP)	New England Biolabs	Ipswich, Massachusetts, USA	Cat#M0290S
Nissl Staining Solution	Sangon Biotech	Shanghai, China	Cat#E607316
Hematoxylin-Eosin(HE) staining kit	Sangon Biotech	Shanghai, China	Cat#E607318

**Antibodies**			
**REAGENT or RESOURCE**	**SOURCE**	**LOCATION**	**IDENTIFIER**
Rabbit polyclonal antibody anti-TTC36	produced previously		DOI: 10.1016/j.gep.2016.11.001
Rabbit polyclonal antibody anti-HPD	Abclonal	Wuhan,China	Cat#A6505
Rabbit polyclonal antibody anti-HPD Phospho T382	Signalway Biotechnology	Pearland, Texas, USA	
Rabbit polyclonal antibody anti-PELI1	Cell Signaling Technology	Maryland,USA	Cat#31474S
Rabbit polyclonal antibody anti-STK33	Cell Signaling Technology	Maryland,USA	Cat#95343S
Mouse monoclonal antibody anti-HA	Santa Cruz Biotechnology	Texas,USA	Cat#sc-7392
Mouse monoclonal antibody anti-Ub	Santa Cruz Biotechnology	Texas,USA	Cat#sc-8017
Mouse monoclonal antibody anti-Flag	Sigma-Aldrich	Missouri, USA	Cat#F3165; RRID: AB_259529
Mouse monoclonal antibody anti-His	Sigma-Aldrich	Missouri, USA	Cat#SAB2702219
Mouse monoclonal antibody anti-β-actin	Sigma-Aldrich	Missouri, USA	Cat#A1978
Mouse monoclonal antibody anti-β-tubulin	Sigma-Aldrich	Missouri, USA	Cat#T5201
Rabbit polyclonal antibody anti-GAPDH	Sigma-Aldrich	Missouri, USA	Cat#SAB2701825
Rabbit polyclonal antibody anti-GFAP	Sigma-Aldrich	Missouri, USA	Cat#SAB4501162
Mouse monoclonal antibody anti-Phospho-Threonine	Cell Signaling Technology	Maryland,USA	Cat#9386S
Anti-Rabbit IgG (H+L) 568 antibody	Sigma-Aldrich	Missouri, USA	Cat#SAB4600310
Goat Anti-Rabbit IgG, HRP Conjugated	CWBIO	Beijing, China	Cat#CW0103S
Goat Anti-Mouse IgG, HRP Conjugated	CWBIO	Beijing, China	Cat#CW0102S
Rabbit polyclonal to GFP - ChIP Grade	Abcam	Massachasetls,USA	Cat#ab290

**Plasmids**		
**REAGENT or RESOURCE**	**SOURCE**	**LOCATION**
pcDNA3.1-TTC36	this paper	
pcDNA3.1-His/V5-HPD	this paper	
pcDNA3.1-His/V5-HPD T2A	this paper	
pcDNA3.1-His/V5-HPD T3A	this paper	
pcDNA3.1-His/V5-HPD T23A	this paper	
pcDNA3.1-His/V5-HPD T138A	this paper	
pcDNA3.1-His/V5-HPD T219A	this paper	
pcDNA3.1-His/V5-HPD T271A	this paper	

pcDNA3.1-His/V5-HPD T337A	this paper	
pcDNA3.1-His/V5-HPD T382A	this paper	
CMV-Flag-HPD(1-280aa)	this paper	
CMV-Flag-HPD ΔC(1-165aa)	this paper	
CMV-Flag-HPD ΔN(166-393aa)	this paper	
CMV-Flag-HPD ΔN1(166-280aa)	this paper	
CMV-Flag-HPD ΔN2(281-393aa)	this paper	
CMV-Flag-HPD VOC2(180-338)	this paper	
CMV-Flag-HPD deletion of ΔTPR binding site	this paper	
CMV-Flag-PELI1	this paper	
CMV-Flag-PELI1 R104A	this paper	
pcDNA3.1-STK33	this paper	
pcDNA3.1-STK33 K145M	this paper	
pTriEX-HA-ubiquitin	this paper	
pGIPZ-Ttc36-shRNA#1	GE Dharmacon	Lafayette, Colorado, USA
pGIPZ-Ttc36-shRNA#2	GE Dharmacon	Lafayette, Colorado, USA
pGIPZ-Peli1-shRNA	GE Dharmacon	Lafayette, Colorado, USA
pGIPZ-Stk33-shRNA#1	GE Dharmacon	Lafayette, Colorado, USA
pGIPZ-Stk33-shRNA#2	GE Dharmacon	Lafayette, Colorado, USA

**Cell Lines**	
**REAGENT or RESOURCE**	**IDENTIFIER**
LO2	Lab reserved
HEK293T	Lab reserved
**Bacterial**	
**REAGENT or RESOURCE**	**IDENTIFIER**
DH5α	Lab reserved
Bl21(DE3)	Lab reserved

Supplementary Table 2. Target sequences used for shRNA construction.

Primer Name	Sequence
h-Ttc36 shRNA#1	5'-TCTTCTTCTCGTTCTTCCT-3'
h-Ttc36 shRNA#2	5'-TGTTGTAGGCTGAAGCCCT-3'
h-Peli1 shRNA	5'-TAACGAGCCATCTTGTAAC-3'
h-Stk33 shRNA#1	5'-TTAGCAGCAAGTTAATGGC-3'
h-Stk33 shRNA#2	5'-TAGATTTCCTCAATAG CAG-3'

Supplementary Table 3. Primers used for quantitative PCR.

Primer Name	Sequence
F-m-Hpd	5'-GCACACGGAGTACAGCTCTC-3'
R-m-Hpd	5'-GGCGGATTGCTGTGATGATG-3'
F-m-Ttc36	5'-CCCTGGAGTTGCAGGGAGTG-3'
R-m-Ttc36	5'-CCAGGGCGCCTGCTACATC-3'
F-m-18S	5'-GTAACCCGTTGAACCCCATT-3'
R-m-18S	5'-CCATCCAATCGGTAGTAGCG-3'
